# Lessons learned from a qualitative evaluation of Veterans’ experience with the VA tele-PAP program during the COVID pandemic

**DOI:** 10.3389/frhs.2026.1760059

**Published:** 2026-06-02

**Authors:** Kyu Seo Kim, Ritchie Verma, Alexander Gomez, Annette M. Totten, Katherine Malcolm, Elena Kennedy, Astrid Carrion-Rodriguez, Christopher Cheng, Angela K. Combe, Rebecca M. Jungbauer, Michael Mitchell, Diane Lee, Jennifer L. Martin, Armand M. Ryden, Connor Smith, Kathleen F. Sarmiento, Eilis Boudreau, Michelle R. Zeidler

**Affiliations:** 1 Division of Informatics, Clinical Epidemiology and Translational Data Science, Oregon Health & Science University, Portland, OR, United States; 2 Department of Medicine, San Francisco VA Healthcare System, San Francisco, CA, United States; 3 San Francisco VA Healthcare System, San Francisco, CA, United States; 4 Division of Pulmonary, Critical Care, Sleep Medicine, & Allergy, University of California, San Francisco, CA, United States; 5 Division of Pulmonary, Critical Care, and Sleep Medicine, David Geffen School of Medicine, University of California, Los Angeles, CA, United States; 6 Division of Pulmonary, Critical Care, and Sleep Medicine, VA Greater Los Angeles Health Care System, Los Angeles, CA, United States; 7 Geriatric Research, Education and Clinical Center, VA Greater Los Angeles Healthcare System, Los Angeles, CA, United States; 8 Department of Cellular and Molecular Medicine, Herbert Wertheim College of Medicine, Florida International University, Miami, FL, United States; 9 Geriatric Research, Education and Clinical Center, VA Miami Healthcare System, Miami, FL, United States; 10 VA Portland Health Care System, Portland, OR, United States; 11 Department of Neurology, Oregon Health & Science University, Portland, OR, United States

**Keywords:** COVID pandemic, obstructive sleeep apnea, patient experience, positive airway pressure, qualitative, sleep, telehealth, veterans' experiences

## Abstract

**Introduction:**

Obstructive sleep apnea (OSA), a disorder caused by repeated collapse of the upper airway resulting in pauses in breathing, is highly prevalent among United States (US) Veterans. Prior to the COVID pandemic, telehealth for clinical appointments and diagnostic testing for OSA was used within the U.S. Department of Veterans Affairs (VA); however, experience was limited with telehealth for initiation of PAP (Positive Airway Pressure), mailing the device to the Veteran, and educating on use through a phone or video visit. Limited literature is available evaluating patients’ experience with this “tele-PAP” setup. The aim of this study is to understand the experiences of Veterans with OSA with Tele-PAP or face-to-face PAP setup (“FTF-PAP”) in the US using qualitative methods.

**Methods:**

We conducted semi-structured phone interviews with a convenience sample of 40 Veterans. Interviews included six domains: delivery of PAP device, knowledge of OSA, equipment setup process, challenges using the device, preferred setup modality, and suggestions for improvement. Four investigators coded transcripts using qualitative analysis software. Themes were identified through iterative discussion and consensus. Each transcript was independently coded by at least two coders, and illustrative quotes were identified.

**Results:**

Forty Veterans with an even mix of PAP initiation modalities (Tele-PAP; FTF-PAP) were interviewed. While both delivery and training modalities were generally well received, we identified several patient factors associated with a preference for one modality over the other. These included patients’ preferred learning style, the cost of traveling to the clinic, preexisting otolaryngological problems, and concurrent psychological factors such as post-traumatic stress disorder (PTSD) and claustrophobia. Regardless of modality, many Veterans had trouble recalling who to contact for troubleshooting or refills.

**Discussion:**

Based on Veteran interviews, tele-PAP initiation to treat OSA improved accessibility to sleep medicine and reduced costs for Veterans. Successful PAP therapy initiation depends on several factors that should be identified early in the PAP initiation process to enable selection of the optimal mode of care (Tele-PAP vs. FTF-PAP). Our qualitative data, while limited in number and focused on the Veteran population, document patients’ acceptance of tele-PAP and illustrate that the approach is a viable alternative to F2F-PAP.

## 1 Introduction

Obstructive sleep apnea (OSA) is a disorder caused by repetitive collapse of the upper airway during sleep. Untreated OSA is associated with cardiovascular (1, 2) and cerebrovascular (3) morbidities, anxiety and depression (4), and cognitive deficits (5, 6). Positive airway pressure (PAP) therapy has been shown to significantly improve OSA symptoms and quality of life across a range of disease severities (7). It is a standard of care for OSA as recommended by the American Academy of Sleep Medicine (8).

OSA is most common among older men (9) and prevalent among United States (US) Veterans; 22.2% of the population served by the US Department of Veterans Affairs (VA) in 2018 had diagnosed sleep-related breathing disorders (10). As the VA is responsible for providing lifelong benefits and healthcare to Veterans (11), the demand for VA sleep care services is rapidly rising with VA sleep medicine appointments increasing 16% per year on average between 2013 and 2018 and the average wait time for an appointment is exceeding 40 days according to an analysis of the VA Corporate Data Warehouse (the repository for the Veterans Health Administration clinical data) (12).

Telehealth, the use of electronic and telecommunication technologies to support long-distance clinical healthcare (13), has been associated with better access, reduced wait times, improved patient outcomes and satisfaction, as well as cost-effectiveness (14–18). In response to the unmet demand for sleep apnea-related services, the VA Office of Rural Health funded an enterprise-wide initiative to implement a Tele-Sleep program using tools such as home sleep apnea testing (HSAT), video visits with the Veteran at home, remote patient monitoring, and communication (12, 19) with reported success in various regions (14, 20–22).

The COVID-19 pandemic and widespread requests by health authorities for people to remain at home and avoid unnecessary travel (23) provided an unforeseen impetus to expand telehealth services to include remote initiation of PAP (tele-PAP). In addition, because PAP devices are aerosol-generating and because there was a shortage of personal protective equipment early in the COVID pandemic, sleep services, including PAP setup, were halted at the VA. Tele-PAP includes delivering the PAP to the patient (usually by mail, but occasionally by patient pickup) and then providing PAP education via a remote modality, such as a phone call or video visit.

Expanding telehealth to include Tele-PAP supports the Veterans Health Administration's mission to provide exceptional care and its commitment to improving access for Veterans (24). Beyond VA healthcare services, Tele-PAP also aligns with patient-centered care models that incorporate patient experience and PREMS (patient-reported experience measures), which often focus on improving access to and ease of use of healthcare services (25, 26).

Recent studies on patients' views of telehealth for OSA care, including remote monitoring and telemedicine visits, indicate that it is generally a positive experience and is linked to better outcomes (19, 27). However, there is limited qualitative research exploring patients' experiences with tele-PAP. The aim of this study is to understand the experiences of Veterans with OSA with Tele-PAP or FTF-PAP in the US, using qualitative methods.

## 2 Methods

A convenience sample of forty patients was selected from a larger cohort (*n* = 435) of patients with OSA referred for FTF-PAP vs. tele-PAP (28). In the FTF-PAP setup, the patient physically came to the sleep center and was instructed on how to use the PAP. In the tele-PAP setup, the PAP was mailed to the patient's home, and the patient was then instructed in its use via a phone call or a video visit. The larger cohort study evaluated for non-inferiority of PAP compliance and efficacy between the groups. The larger cohort study and this qualitative study were deemed research-exempt quality improvement (QI) projects by the San Francisco and Greater Los Angeles VA IRBs.

For this study, patients referred for PAP from 2019 to 21 were interviewed by telephone in 2023. Per the inclusion criteria, only individuals with new PAP starts at VA were included. Two patients within the sample were later found to have prior experience with PAP therapy prescribed by non-VA healthcare providers, leading to the inclusion of two patients with prior PAP experience. The Veterans were informed that participation in the interview was voluntary and for quality improvement purposes (i.e., non-research purposes). They were also informed that the conversation would be transcribed, remain confidential, and not affect the care they received. Written informed consent was not required due to the QI nature of the study. Following verbal consent, four interviewers (Greater Los Angeles: ACR, CC; San Francisco: KM, EK) conducted a semi-structured interview using an interview script (Supplementary Table 1) developed by evaluation contractors informed by their experience in patient engagement and in consultation with VA sleep program leadership and clinicians. The interview was designed to cover six domains: delivery of PAP device, knowledge of OSA and PAP, equipment setup, challenges using PAP therapy, preference for setup modality, and suggestions for improvement. Each interview was expected to last 15–20 min. The interviews were transcribed using Microsoft Teams and reviewed for accuracy by two investigators (RR, KK) by comparing them with the interviewer's notes. Four researchers who were not interviewers (RV, KK, AC, and AT) developed an initial list of codes based on the interview domains, then revised and expanded these codes after reviewing a subset of the transcripts. Coding was then completed by the four researchers, with at least two coders independently reviewing and coding each transcript to ensure reliability and consistency. The coded transcripts were analyzed using thematic analysis, and illustrative quotes were identified for each theme. Disagreements regarding coding, naming themes, or quote selection were resolved through discussion and consensus. We focused on what was well-received and what was not in each step of the PAP delivery, set-up, and ongoing usage, with attention to how Tele-Pap and FTF-Pap compared, as well as each Veteran's preference if they were given the opportunity to repeat this process, given that this was part of a quality improvement project. The qualitative coding and summarization were conducted using a software tool, Dedoose 9.0.107^TM^.

This study was evaluated by the VA Greater Los Angeles Institutional Review Board (IRB) and the San Francisco VA IRB, and both determined that the project met the definition of quality improvement (not research). VA's Virtual Care Core provided funding for the project.

## 3 Results

Forty Veterans with OSA who initiated PAP treatment during the cohort recruitment period completed the interviews (Table 1). The participants included 20 Veterans from the San Francisco area and 20 from the Greater Los Angeles area and were predominantly male (95%) and White (65%).

**Table 1:** Characteristics of the study participants.

Characteristic	*N* = 40
Age	
Mean (SD)	58 (16)
Median (Q1, Q3)	58 (48, 73)
Min, Max	31, 85
Gender, *N* (%)	
MALE	38 (95%)
FEMALE	2 (5.0%)
Race, *N* (%)	
White	26 (65%)
Black or African American	5 (13%)
Asian	2 (5.0%)
Native Hawaiian or Other Pacific Islander	2 (5.0%)
American Indian or Alaska Native	1 (2.5%)
Unknown	4 (10%)
Ethnicity, *N* (%)	
Hispanic or Latino	6 (15%)
Not Hispanic or Latino	31 (78%)
Unknown	3 (7.5%)
Marital Status, *N* (%)	
Never Married	11 (28%)
Married	20 (50%)
Separated	2 (5.0%)
Divorced	7 (18%)
Widowed	0 (0%)
Unknown	0 (0%)
Rurality, *N* (%)	
Urban	31 (78%)
Rural	9 (23%)
Distance, miles^1^	
Mean (SD)	75 (84)
Median (Q1, Q3)	48 (16, 115)
Min, Max	3, 369
Unknown	5
Charlson Comorbidity Index	
Mean (SD)	2.43 (2.37)
Median (Q1, Q3)	2.00 (0.00, 4.00)
Min, Max	0.00, 10.00

N, numbers; SD, standard deviation; Q1, 1st Quartile; Q3, 3rd Quartile.

1 Great Circle Distance to Greater Los Angeles VA or San Francisco VA (depending on where the Veteran resided).

Table 2 provides information on how the PAP device was delivered and how set-up instructions and education were provided. Eighteen Veterans received their instructions (how to use and maintain the device) face-to-face (FTF-PAP). Of these, 17 received their device in person, and one had it mailed to them. On the other hand, 20 Veterans received their instructions remotely (12 by phone and 8 via synchronous video), both of which were considered Tele-PAP. All in this group had their devices delivered via mail, except one Veteran who picked it up in person. Two Veterans declined to receive instructions because of their prior experience with PAP, who had received their devices by mail. Although the intent of the study was to include individuals with new PAP starts, patients who had initiated PAP outside of the VA were included.

**Table 2:** Delivery modality of the PAP device and mode of the setup instructions.

Setup instructions	Delivery modality of PAP device	
	In Person	Mailed or shipped
FTF-PAP	17 (8 SF, 9 GLA)	1 (1 SF)
Tele-PAP (Remote, video)		8 (8 SF)
Tele-PAP (Remote, phone)	1 (1 GLA)	11 (1 SF, 10 GLA)
None (declined by patient)		2 (2 SF)

FTF-PAP, face-to-face (FTF) Positive airway Pressure (PAP) generally includes receiving the device and instructions FTF; Tele-PAP, generally, includes receiving the device by mail and instructions via either phone or video; GLA, Greater Los Angeles; SF, San Francisco.

Table 3 lists the categories that emerged from our analysis. These categories include their experiences with getting their devices FTF vs. remotely, and how Veterans were initially trained on the use of the PAP device. Furthermore, factors emerged during these interviews, such as patient convenience, learning style, prior knowledge of sleep apnea, mask fit, and self-reported factors related to device use, remote patient monitoring, and access to supplies, which could have impacted their experience with FTF vs. Tele-PAP setup and maintenance. These categories, along with the themes, are described in more detail in the following sections as results.

**Table 3:** List of the categories from qualitative analysis.

Themes
1. Experience with Device Delivery
2. Experience with Initial Training
3. Factors that impacted experience a. Learning style b. Convenience c. Mask fit d. Ability to access refills and troubleshooting support e. Knowledge about Sleep Apnea f. Benefits of Remote patient monitoring
4. Factors that impacted self-reported use of PAP therapy

PAP, positive airway pressure.

### 3.1 PAP device delivery and initial training

Participants reported that PAP device delivery was generally problem-free, regardless of the instruction method (FTF-PAP or tele-PAP). All but one Veteran [Patient SF17 received the device and setup instructions in person but had a poor experience with a “bad receiver” (an unspecified part of the device) that needed to be exchanged] received fully intact new equipment in excellent condition. There were some complaints about the courier service, including delivery delays and leaving packages unattended without requiring the recipient's signature. Most Veterans reported positive experiences learning how to set up, operate, and maintain the devices. Select illustrative comments are quoted below with the Veteran's setup modality given in parentheses. A Veteran described a typical FTF-PAP delivery and setup:

Patient GLA1 (FTF-PAP): “At the hospital, we walked through all the steps, he (trainer) fitted me for a mask, so I knew exactly which one to use as we went through it all, and he also told me to use distilled water. […] he also told me how often to replace the filters on it and how to keep it all clean. He walked through that and how to wash the hose.”

“He went through the fitting of the mask and how to fit it properly.”

Interviewer CEC: “And did you find your questions were answered and you have contact like if you had any additional questions, did you know who to contact?”

Patient GLA1: “Yes, he gave me his card.”

Another Veteran described a typical remote (tele-PAP) delivery and setup

Patient GLA2 (tele-PAP): “I think I just read it and understood, and that it was preset to a pressure, so I didn't have to do anything much more than just turn it on, put the filters and stuff in it and you know, and just address the straps on my head.”

### 3.2 Patient learning style

There was a large variability in Veterans’ preferences for FTF-PAP vs. tele-PAP training, depending on their communication and learning styles. The Veterans who preferred FTF-PAP training cited their hands-on learning style uniformly as the most important reason:

Patient GLA3 (tele-PAP): “I just do better with hands on.”

Patient GLA4 (FTF-PAP): “You're right there with somebody, you can actually, like, physically touch the device.”

Patient SF1 (FTF-PAP): “[…] in case you miss anything or have any questions, I mean, you're right there.”

Patient GLA5 (FTF-PAP): “Because I get better understanding in person than any other way.”

Patient SF2 (FTF-PAP): “It sinks in better.”

Patient GLA6 (FTF-PAP): “I'm deaf in one ear so I have to read lips. So, it's easier if I do it in person.”

Other reasons for preference for *FTF-PAP* training included the perception of a more personalized experience and ease of asking questions:

Patient SF3 (FTF-PAP): “[…] the information that was provided was more directed at me personally and my specific needs.”

Patient SF4 (FTF-PAP): “Because when I'm talking to somebody, I don't know, I'm more inclined to maybe ask questions that I wouldn't normally do on video.”

However, in contrast to the above perspective, one Veteran felt pressured to ask all their questions on the spot (Patient SF5, *FTF-PAP*) during the FTF training. The Veteran felt all the questions were answered “at the time,” but more questions inevitably came up that the Veteran “didn’t know how to ask.” Another unique challenge with *FTF-PAP* training came from the difficulty in retaining the information taught in the clinic:

Patient GLA7 (FTF-PAP): “… But then I got home. I'm an old fart and I do pay attention, but a lot of times I take my wife …I don't remember it, but it was explained very well. It was just between LA and home. My brain was everywhere else. And I probably didn't pull up right. […] but the person whether is a doctor or tech or whoever, each section was very thorough, and I thought he did a really good job. I didn't have any questions, just I didn't remember everything, probably.”

The above quote highlights that even *FTF-PAP* training needs to be accompanied by easy-to-follow reference material. A Veteran who was trained in person (Patient SF6, *FTF-PAP*) asked for “simpler directions”, “something that you wouldn’t forget too much.” On the other hand, the Veterans who were satisfied with the remote setup found the machine itself or the included instructions self-explanatory and not technically difficult:

Patient GLA8 (tele-PAP): “I mean, they're really easy to use and then it works so well.”

Patient SF7 (tele-PAP): “When I look at the CPAP machine, it's all in there pretty much. When you play with it, it'll let you know how you set it up.” “And they also showed me that it's already set up when they send it to me.”

Patient SF8 (tele-PAP): “Only the phone was fine. The machines are not really that complicated, but the settings on it, they did that through the computer at the hospital. So, I didn't have to make any adjustments, and the instructions were pretty clear. It was pretty easy.”

Patient GLA9 (FTF-PAP): “[…] it's not a pretty difficult thing to put on you know. There you connect the hoses to the end of the machine. Plug it in.”

Veterans with prior PAP experience found the instructions simple and often did not need additional help. This suggests that Veterans who have used PAP before were more likely to choose tele-PAP setup options. However, it was clear that no single solution could satisfy every Veteran's communication and learning style preferences. Between phone and video, however, video was generally preferred by new users:

Patient SF10 (tele-PAP): “If I had a preference or recommendation, I would say in person or video would be OK, but I don't think the phone would be as effective as video.”

### 3.3 Patient convenience

Scheduling convenience and travel cost savings were by far the most essential factors for those who preferred a tele-PAP setup, especially if they did not find the device setup technically too difficult:

Patient GLA9 (FTF-PAP): “Because the traffic is very hard in the morning, you know, […] not to mention the expense of the gas.”

Patient SF11 (tele-PAP): “I would prefer virtually because of my schedule.”

Patient GLA10 (tele-PAP): “Over the phone would have been fine for my distance that I live, yeah.”

One Veteran (Patient SF13, tele-PAP*)* lived six hours away from the nearest VA with a Sleep Center. Another Veteran summarized, weighing the pros and cons:

Patient GLA7 (F2F PAP): “So in-person seems to be more personal, but either way I'd end up going “ohh. I should have asked this!” or whatever. That's just how I am. And I am not a techie, but if you give me a button to push so that we could have the face-to-face Zoom thing or whatever […] there really shouldn't be much difference between [that] and in person, which involves driving to LA in that traffic. Ugh, it's terrible.”

It is important to note that no Veteran in our cohort reported technical difficulties, such as unstable internet or poor phone connections, that could have prevented effective tele-PAP training.

### 3.4 Factors for self-reported usage

The greatest contributor to not using the machine was difficulty keeping the mask on, often related to sleeping habits. Many patients, such as Patient SF6 (FTF-PAP), who tossed and turned, thought “[PAP] just didn’t seem to be compatible with [the Veteran's] sleeping habits”.

Patient SF1 (FTF-PAP): “My bigger issue is I move in my sleep a lot. No mask would ever stay on, nor the piece that goes in your nose. It would never stay on.”

Interviewer KM: “Could we have done anything differently to help you wear it?”

Patient SF1: “No. Maybe if you put a bunch of duct tape around it so it didn't come off my face.”

The above patient had received their PAP devices and training in person. Our analysis revealed that the psychological effect of a tight-sealing mask must be a key consideration in prescribing PAP to Veterans.

Patient SF14 (tele-PAP): “Falling asleep with [PAP] on isn't a big problem, but at night and I don't know if it's because of my PTSD as well, but I rip it off my face so there's it's rarely that I get a full night sleep with it, but there's been times where I've gotten full night sleeps.”

Patient SF7 (tele-PAP): “[It feels] like I'm running out of breath. What you call that, a claustrophobic? When I wear the full-face mask, I started to wake up in the middle the night like I'm running out of air. It's all psychological. [I am] using the one with the nasal only. I'm just scared of it.”

One patient (SF15, tele-PAP), who received the device remotely but ultimately could not tolerate it due to claustrophobia, thought that *FTF-PAP* training and even a stay in the sleep lab might have given him more confidence to use the therapy.

Patient SF15: “[…] would be nice to like, I don't know, this sounds weird, but spend the night and make sure that everything I'm doing it right.”

### 3.5 Mask fit and tolerance

The mask is a critical interface between the nose and/or mouth and the PAP device, and poor mask fit is one of the most common reasons for difficulty using PAP therapy, often requiring a trial-and-error approach to find optimal fit and comfort (29, 30). Therefore, we wanted to explore the impact of tele-PAP mask fit on users.

Patient GLA11 (tele-PAP): “The mask didn't seem like it would fit correctly, and then I would always seem to have air leaks all through the night. And so, it would be really, really uncomfortable.”

Patients appeared satisfied with the tele-PAP setup, as long as they received a quick response from the medical team to identify the problem and send a better-fitting mask.

Patient GLA8 (tele-PAP): “The first one I tried to do was a nose cup, but yeah, I ended up changing after it was not fitting that good and that's the only challenge I had. […] When I called, they said they would send me like the little nose pieces and that came really quick, and I haven't had a problem.”

However, one of the patients (SF15, *tele-PAP*), whose care was significantly delayed due to dental issues, might have been identified earlier with an *FTF-PAP* setup.

Beyond the initial fitting, there were patients with nasopharyngeal or eye issues worsened by PAP treatment, who belonged to either modality and would need interdepartmental coordination: frequent nosebleeds worsened by PAP (Patient GLA14, *tele-PAP*), sinus problems (Patient GLA7, *FTF-PAP*), drying of eyes when the patient had only one functioning eye (Patient SF16, *tele-PAP*). Furthermore, adjusting to and tolerating PAP treatment appeared to be equally challenging for both the *FTF-PAP and tele-PAP* groups.

Patient SF17 (FTF-PAP): “The mask is terrible to wear. I tried everything. I tried every one of that and there was only one of them I could wear but I move, I move so much at night.”

Patient SF4 (FTF-PAP): “There was a little bit of getting used to. Because I'm a mouth breather, so I have to use a full-face mask. And so, I got this big thing set on my head. So yeah, it was a little bit of a challenge to get used to it, but it didn't take long.”

Patient GLA14 (tele-PAP): “[…] they give me a nose cup, right? So, I told him, “Well, you know, my mouth opens up at night.” So, then they gave me a chin strap. […] and using that stuff is like putting a football helmet. It's just so much stuff you have to do, and I don't like that. And I have a beard and mustache. So, I just wish it was something that would just cover my nose and mouth everything make life a lot easier for me.”

Patient SF18 (tele-PAP): “I found getting used to the mask very difficult, and sometimes I wake up in the middle of the night, my mask was pulled off a couple times. It took me a while to figure out how to adjust it properly through. It wasn't annoying and it fit fine and that took a while. Took me about six months to get used to it, and after two years, I'm still not quite used to it.”

### 3.6 Reaching troubleshooting support and obtaining refills

A common issue for both *FTF-PAP* and tele-PAP was that patients were not always sure who to contact when they experienced difficulties (troubleshooting), such as a mask leak or questions. Some patients could not recall whether they had been given contact information, even when protocols were in place to ensure it was provided. This was a critical issue for the population of Veterans with OSA, who tended to be older, and some had cognitive problems.

Patient GLA15 (tele-PAP): “In the first three months I did not [know who to contact]. I don't know if that was my fault or if that was just not given to me ever, since I have not had an issue.”

Patient GLA12 (tele-PAP): “[…] there was never any support number for the CPAP. You just kind of received it and just kind of like, throw it on. But I didn't have anyone. I didn't have a number for questions.”

Veterans' experiences with attempting to reach someone for assistance were highly heterogeneous; some cited ease of access, while others had frustrating experiences.

Patient SF8 (tele-PAP): “Well, the last guy the last time I called, you know, I left a voicemail and I said, hey, I need more filters and I'm having trouble with the hose. And one of the guys called me back and he gave me his direct extension so that I could do that. And then the next time I needed filters and stuff, I called and went through the prompts to his voicemail and just left a message, and the stuff came in the mail. So, you know he got the message, and I got what I needed….”

Patient GLA7 (FTF-PAP): “In the VA system, sometimes it's hard to get to where you wanna go without having to wait for a call back or whatever, but the sleep center's number, there was always someone there that was very helpful.”

Patient GLA13 (tele-PAP): “[T]here are two sleep clinics and one of them is great. One of them was really, really hard to get a hold of. Lines were terrible and […] it was really hard to get ahold of somebody because the phones were always off the hook, or the messages were full. But this was also during COVID, so people were probably slamming their voicemail boxes, but I eventually got referred back to [a different] sleep clinic. And it was just like they waved the wand, and everything just worked, and I got everything I needed.”

The Veterans who used the VA's patient portal My HealtheVet for secure messaging noted satisfaction with the platform, but not as a substitute for communication by phone:

Patient SF19 (tele-PAP): “Yeah, usually what I've been doing recently is, I've been using my HealtheVet app, which has been extremely convenient, and they've been really prompt and responsive. So that's the main source of communication I've been using, which were working great for me. […] But I guess if I just needed to speak with someone at the immediate moment, you know, sometimes it might be difficult to get a hold of somebody.[…]. If you try to call the number there, no one answers, and you have to leave a voicemail.”

The lack of a standardized way for Veterans to obtain refill supplies of consumables appeared to result in heterogeneous experiences as well. Some had a smooth experience, but in a few cases, Veterans struggled due to either not knowing where to contact or inefficiencies and delays in the system.

Patient SF13 (tele-PAP): “Well, the only thing is I have to order once a year or something. […]That's really all I have to do. Yeah, it's very easy.”

Patient GLA16 (tele-PAP): “The only problem I have with the CPAP is that I need the materials, …The machine even came on and told me to change them. It's been well over six months since. […] they're all past due and I don't know where to go to get them.”

Patient GLA12 (tele-PAP): “I had a number to order supplies, but I couldn't order supplies in a month. It had to be like 3 months passed before I could order a supply. I had to leave a message. But then the message wasn't received. So, I had to keep calling back making sure I spoke with someone to make sure that my supplies were actually put into the system and that I received them.”

Patient GLA17 (FTF-PAP): “Maybe they could just automatically send the refills, or you know, stuff like that, extra hoses and all that, because I tried ordering the parts online, but I never got them.”

### 3.7 Impact of remote monitoring

One of the strengths of the Tele-Sleep program, regardless of the setup modality, was the ability for providers to remotely monitor and adjust the PAP device settings. Multiple Veterans expressed a sense of safety from knowing that someone monitored their information via the PAP device (apnea-hypopnea index, mask leak, etc.).

Patient GLA18 (FTF-PAP): “[…] the replacement one I had some issue with how it was set up, and I did contact the VA, and they changed the settings on it remotely.”

Patient SF5 (FTF-PAP): “I had to reach back out because the air was just too strong at times … and I think that's what woke me up. And then we adjusted that (over telehealth).”

Patient SF13 (tele-PAP): “[The training] was on video and they told me and my wife that she can set this up right here, and she will be watching the numbers anyway. So, she set it up and I called and talked to her one time that it was a little too warm, and she had me turn it down a little bit.”

### 3.8 Impact on knowledge about sleep apnea

Veterans' understanding of their sleep apnea diagnosis and potential complications varied.

Patient SF10 (FTF-PAP): “[…] if you have apnea in some fashion, for some reason it can be a contributor to a higher than the healthy blood pressure, so that is the reason why I use the machine.”

Patient SF11 (tele-PAP): “Not only like, is it gonna help me for the fatigue in the day, it'll help my heart condition in the future. Also, there's better sleep. I can help minimize the risk of obesity and diabetes.”

Patient GLA19 (tele-PAP): “So that you can actually get a good night's rest.”

Patient GLA20 (FTF-PAP): “I don’t remember.”

There was no apparent relationship between knowledge about sleep apnea and the way Veterans received instructions on how to use the PAP machine. Also, knowledge about sleep apnea did not appear to be a barrier to usage, nor did it seem to affect their preference for FTF-PAP vs. tele-PAP setup.

### 3.9 Veterans experience

Veterans overwhelmingly endorsed the overall quality of the Tele-Sleep program regardless of how PAP was initiated.

Patient SF16 (tele-PAP): “I don't know if this should be said on a recorded line, but you people are too generous offering masks and things.”

Patient SF12 (FTF-PAP): “A gentleman spent good 45 min on the phone with me getting everything arranged and shipped out. And once I got it here, got all set up. I just hollered back at him. You sent me everything I needed. You did a great job.”

Patient SF20 (tele-PAP): “[…] it was fine. VA did a good job and set everything up. And I'm doing the consult over the video. So, I was happy with how they set it up and everything.”

Patient GLA5 (FTF-PAP): “I'm very happy with the CPAP program and it has helped me. And I would advise anyone that needs one to get it and use it.”

## 4 Discussion

The national VA Tele-Sleep program was initially launched to improve U.S. Veterans' access to sleep medicine services, particularly those Veterans living in rural areas (12). The COVID-19 pandemic significantly changed the landscape of telehealth (31), including that of sleep medicine. Restrictions related to the COVID-19 pandemic led to a shift to tele-PAP distribution and setup, although it was unclear whether this pathway was as effective as a standard *FTF-PAP* setup in terms of patient compliance with therapy and satisfaction with the medical interaction. This is, to our knowledge, the first study to qualitatively evaluate Veteran experience with tele-PAP.

To address the lack of literature regarding Veteran satisfaction with F2F-PAP vs. tele-PAP, we qualitatively evaluated the experience of Veterans establishing PAP care with the VA during the pandemic at two large VA medical centers. We found that while both FTF-PAP and tele-PAP were generally well received, individual factors must be considered to improve patient satisfaction and experience. Beyond the initial setup phase, the issues affecting patients were similar. We gained valuable insights into what may drive patient satisfaction and what could be changed to improve patient experience in this and other programs.

Regular, nightly PAP use is a major undertaking, but it is necessary if the potential benefit of the treatment is to be realized and the investment in equipment and staff justified. Our analysis underscored the importance of a patient's preferred learning style. Veterans who valued hands-on, in-person training for ease of asking questions were best served by FTF. Some Veterans, many of whom were new users of PAP, were comfortable following directions given remotely with a combination of written instructions. Given this observation, it may be helpful to have Veterans view an instructional video and/or written instructions *before* scheduling PAP device delivery so they can decide which training modality would work best for them. Between video and phone, Veterans almost unanimously preferred video. The video was the preferred mode of care delivery; however, early in the pandemic, there were often technical issues with video visit capabilities at the VA, which have since been resolved. However, visual and hearing deficits must be taken into account, and some people will need alternative formats.

The Veterans who preferred tele-PAP training wanted to reduce the time and financial cost of travel, but they still considered the benefits of FTF training. The balance was dependent on the individual's comfort level with following remote or asynchronous instructions. Our analysis of this cohort did not identify internet access as a barrier that limited video calls; nevertheless, this must be considered when deciding on training modality. Of note, the VA provides devices and internet services to Veterans who cannot afford them and offers technical assistance to those who cannot set them up on their own. This makes Veterans' experiences less generalizable to patient groups without access to these resources.

Not surprisingly, finding a properly fitting and tolerable mask was a challenge for both the tele-PAP and FTF-PAP groups. This disadvantage could be mitigated by promptly sending out different masks or using an AI-generated mask-fitting app. Breathing style -nose or mouth breather—should be considered prior to shipping the device to reduce initial challenges. We learned that medical issues that can affect PAP use, particularly otolaryngological and dental problems, should be identified prior to PAP initiation. Psychological factors such as PTSD and claustrophobia, particularly pertinent to Veterans, may be better addressed in person, where an expert can ensure proper fitting and provide reassurance and encouragement. In cases where this is a significant problem, referrals for behavioral health consultation or treatment may be neede d.

Some Veterans did not know where to call for support or supply refills, regardless of the setup and training modality. This needs to be addressed by providing frequent reminders and reinforcing the fact that communication is possible. For example, at every follow-up, a clinician can review the email address to use or phone numbers to call. Every refill delivery could include a slip with information for future contacts. Patient portals, in this case, My Health*e*Ve*t*, can also be utilized to periodically send messages to contacts.

Veterans valued that a clinician could remotely observe their PAP sleep data and adjust treatments as needed. Overall, they reported mostly positive experiences with the Tele-Sleep program. From the patient perspective, telehealth care is generally comparable to or better than in-person visits for many types of appointments, especially regarding convenience and access, as shown in our findings and supported by existing research (32–34).

### 4.1 Limitations

Our interviews were limited to a convenience sample of patients who were willing to be interviewed, and very few women were included; therefore, we cannot speak to possible sex differences in experiences and preferences. The time period between PAP initiation and when the interviews were conducted was less than ideal due to COVID-19 pandemic-related disruptions. We were unable to address recall bias in our analysis. Our findings may not be generalizable to non-Veteran patients or to Veterans receiving care at other VA facilities.

### 4.2 Conclusions

This qualitative analysis of Veterans who initiated PAP treatment for OSA through the VA TeleSleep program demonstrated that, with appropriate preplanning, including discussions regarding the preferred method of education at the time of appointment scheduling, procedures for identifying mask preferences, and clear information on who to contact for support or to refill supplies, Veterans accepted tele-PAP as an option. Given the increased convenience of tele-PAP setups for many Veterans, especially those not living near a VA facility, our findings demonstrate that tele-PAP is a viable alternative to F2F-PAP for many Veterans, and expanding this service is one way to reduce barriers to care for Veterans.

## Data Availability

The datasets presented in this article are not readily available because given the sensitive nature of the personal data collected during this study, the data (transcripts) are not publicly available. Requests for additional information can be made to the corresponding author. Requests to access the datasets should be directed to Kathleen F. Sarmiento; Kathleen.Sarmiento@va.gov.
