# Supplementary material for: Lessons learned from a qualitative evaluation of Veterans’ experience with the VA tele-PAP program during the COVID pandemic
Source: Front Health Serv. 2026 Jun 2;6:1760059. doi: 10.3389/frhs.2026.1760059 (PMC13269075; doi:10.3389/frhs.2026.1760059)
Supplement: Supplementary file 1 [file Table1.docx]

**Appendix A. Script for semi-structured interviews**

**Introduction script (Voicemail)**

Hi, my name is … and I work at the … VA. We are interviewing Veterans as a part of a VA quality improvement project to improve CPAP services for all Veterans with sleep apnea. Participation is voluntary and will not affect your healthcare at VA. The interview should take 15 minutes or less. Please call me back at … if you are interested in speaking. You may also let me know that you are not interested in speaking. I will also call you back in approximately 10 minutes. Thank you, goodbye.

**Introduction script**

Hi, my name is … and I work at the … VA. We are interviewing Veterans to improve CPAP services. Participation is voluntary and will not affect your care at VA. The interview should take 15 minutes or less. Are you willing to talk with me?

No – OK. Thank you for taking the call and for your service. (hang up)

Yes – Thank you. We would like to record this conversation to make a transcript so that we can combine and summarize the answers from all the Veterans we talk to. We will use the recordings only to make the transcript and not for any other purposes. Your name will not be in the transcript or the results. Can I record this?

No – OK. Thank you for taking the call and for your service. (hang up)

Yes – I will call you from a line where I can record out conversation. The area code will be … If you are ready, I will hang up now and call you right back.

No – OK. Thank you for taking the call and for your service. (hang up)

Yes – (call back) This is … calling you back. I want to confirm that I have your permission to record this call.

**Script**

I would like you to focus on the time from when you were first diagnosed with obstructive sleep apnea through the first months of receiving your CPAP equipment.

1. Equipment delivery: Did you receive it in person or by mail? Did you have any difficulty with receiving the device? Was it in good condition with all the parts you needed (hose, mask, power cord, etc.)?
2. CPAP knowledge: Have you been told by a healthcare professional that you have sleep apnea? Can you tell me in your own words what sleep apnea is? Why is it important to treat sleep apnea?
3. Equipment training: How did you receive training on CPAP use from VA? In person, by phone, or video? Did they talk to you about how to clean the device, and what to do if there was a mask leak?
4. Training modality preference: If you had to do it again, would you prefer to be trained in person, by video, or by phone? Why?
5. Follow-up: Did you know who to contact if you had additional questions or problems? Did you ever try to contact anyone with questions or problems?
6. Use difficulties: Overall, what worked well during this process of getting started on CPAP? What challenges did you have? What helped you get over the challenges? (If not using CPAP) What made you stop using CPAP?
7. Additional comments: Is there anything about CPAP and setup that could be improved or changed?

**Closing remarks**

Thank you very much for your time. We really appreciate your willingness to help us provide better care to Veterans. Goodbye.
